# Comparison of Depth-Related Visuomotor Task Performance in Uniocular Individuals and in Binocular Controls With and Without Temporary Monocular Occlusion

**DOI:** 10.1167/iovs.65.8.32

**Published:** 2024-07-19

**Authors:** Preetirupa Devi, Joshua A. Solomon, Christopher W. Tyler, Tarjani V. Dave, Swathi Kaliki, Shrikant R. Bharadwaj

**Affiliations:** 1 Centre for Applied Vision Research, City, University of London, London, United Kingdom; 2 Brien Holden Institute of Optometry and Vision Sciences, L V Prasad Eye Institute, Hyderabad, India; 3 Brien Holden Eye Research Centre, L V Prasad Eye Institute, Hyderabad, India; 4 Hariram Motumal Nasta and Renu Hariram Nasta Ophthalmic Plastic Surgery Service, L V Prasad Eye Institute, Hyderabad, India; 5 Operation Eyesight Universal Institute for Eye Cancer, L V Prasad Eye Institute, Hyderabad, India

**Keywords:** binocular advantage, head movements, motion parallax, retinoblastoma, uniocular vision, visuomotor

## Abstract

**Purpose:**

Do one-eyed (uniocular) humans use monocular depth cues differently from those with intact binocularity to perform depth-related visuomotor tasks that emulate complex activities of daily living? If so, does performance depend on the participant's age, duration of uniocularity and head movements?

**Methods:**

Forty-five uniocular cases (age range 6–37 years; 2.4 months–31.0 years of uniocularity) and 46 age-similar binocular controls performed a task that required them to pass a hoop around an electrified wire convoluted in depth multiple times, while avoiding contact as indicated by auditory feedback. The task was performed with and without head restraint, in random order. The error rate and speed were calculated from the frequency of contact between the hoop and wire and the total task duration (adjusting for error time), respectively, all determined from video recordings of the task. Head movements were analyzed from the videos using face-tracking software.

**Results:**

Error rate decreased with age (*P* < 0.001) until the late teen years while speed revealed no such trend. Across all ages, the error rate increased and speed decreased in the absence of binocularity (*P* < 0.001). There was no additional error reduction with duration of uniocularity (*P* = 0.16). Head movements provided no advantage to task performance, despite generating parallax disparities comparable to binocular viewing.

**Conclusions:**

Performance in a dynamic, depth-related visuomotor task is reduced in the absence of binocular viewing, independent of age-related performance level. This study finds no evidence for a prolonged experience with monocular depth cues being advantageous for such tasks over transient loss of binocularity.

Our ability to compute distance, depth, and curvature information from two-dimensional monocular retinal images is enabled through a combination of binocular retinal disparity and several monocular cues to depth (e.g., motion parallax, linear perspective, occlusion, shading, shadows, etc.).^1^^–^^4^ Individuals with degraded binocularity, such as in amblyopia and strabismus, are noted to have reduced depth perception from disparity.^5^^–^^8^ This deficiency is also reflected in the depth-related visuomotor tasks like placing pegs on a pegboard or placing beads on a needle.^5^^,^^6^^,^^9^^–^^11^ In these situations, the integration of the rudimentary binocular disparity cue and the monocular depth cues might impoverish the cumulative depth estimations during the visuomotor task.^4^ Therefore ascertaining the contribution of monocular depth cues to the visuomotor task performance, independent of binocular disparity's influence, may not be possible for these individuals. Individuals with only one functional eye (henceforth referred to as uniocular) are completely devoid of binocular cues and the aforementioned judgments become entirely dependent on monocular cues. These individuals also have the benefit of estimating depth exclusively from monocular cues over a long period of time, and they might develop strategies to overcome the challenges posed by the absence of retinal disparity. Gonzalez et al.^12^ observed that, compared to binocular viewing, uniocular children and those with one eye occluded temporarily were equally poor at estimating the relative depth between the two rods of a Howard-Dolman–type depth estimation apparatus. Although this study implies suboptimal depth perception after loss of vision in one eye and limited long-term compensation for this vision loss, the results may be specific to the impoverished cues of the experimental apparatus and may not generalize to more complex real-world visuomotor activities of daily living where multiple monocular depth cues are available.^12^ Considering this gap in the literature, for the present study we asked two fundamental questions related to the functional depth vision of uniocular individuals: is the performance of uniocular individuals in a visuomotor task requiring accurate depth perception equivalent to fully binocular individuals under monocular viewing conditions? If not, is uniocular task performance dependent on age, duration of uniocularity, or the use of head movements that should provide monocular depth cues such as motion parallax or occlusion?

The answers to these questions may be task dependent.^13^ Tasks that are “easy” to perform may not be able to differentiate uniocular/monocular performance from binocular performance owing to a ceiling effect. On the other hand, tasks that are too “difficult” to perform may result in similarly indistinguishable outcomes owing to a floor effect. Therefore, it is critical to choose a task that can reliably distinguish the performance change arising from the loss of binocularity in the visual system. The functional depth-vision task used in this study—a buzz-wire task—was inspired by the study of Read et al.^14^ that showed a significant difference in task performance of visually healthy adults under binocular and monocular viewing conditions. The buzz-wire task requires the participants to pass a hoop around a convoluted wire track set in depth, without touching the wire (Fig. 1A). Physical contact between the hoop and the wire results in an auditory “buzz,” signaling an error in the task. The participant's goal is to traverse the length of the wire as quickly as possible while making as few errors as possible. Read et al.^14^ observed that the number of errors and the total time taken to complete the task were lower under binocular than monocular viewing conditions. This binocular advantage potentially arises from enhanced estimates of the wire's slant, curvature, and diastereopsis (ability to perceive the gap between two surfaces) using the several depth cues that are consistent with each other and combined in a statistically optimal fashion during natural viewing.^4^ Similar tasks involving the buzz-wire apparatus have also been used by Joy et al.^15^ and Murdoch et al.^16^ for investigating the impact of degraded binocularity on visuomotor performance, albeit more qualitatively than Read et al.^14^

**Figure 1.**
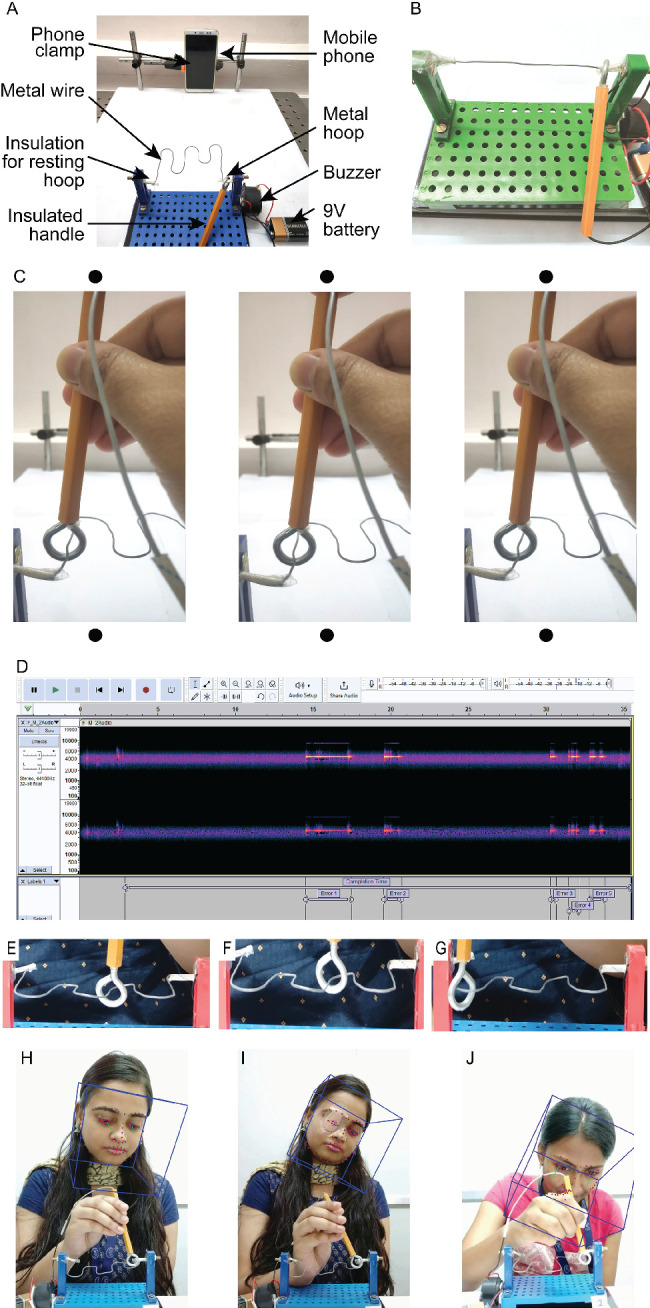
(**A**) The buzz-wire experimental setup as viewed by the participant with the key elements highlighted. (**B**) The unbent buzz-wire used in this study. (**C**) A representative fusable stereoscopic photograph depicting the position of the metal hoop and the wire track in depth. The eyes may be converged to fuse the left and middle panels or diverged to fuse the middle and right panels to perceive depth in the stereogram. (**D**) A representative screenshot of a spectrogram obtained using the Audacity software, with labels marked for the completion time and for the epochs of error time-stamps (high-contrast tracks in the spectrogram) during a representative trial. (**E**–**G**) show representative photographs of the hoop position during the straight (**A**), transition (**B**), and curved (**C**) portions of the wire track. (**H**–**J**) Representative frames of head tracking performed by the OpenFace software used in this study.^17^ (**H**, **I**) Frames from the binocular and monocular viewing conditions, respectively, with successful head tracking, and (**J**) a frame from a discarded video where the head tracking failed. Additional consent was obtained from the participants shown above to use their pictures in this figure.

The following hypotheses were formulated to address the questions raised earlier. First, the increased variability of depth estimates in the absence of binocularity may result in a greater number of errors in the buzz-wire task in uniocular individuals and in controls under monocular viewing. The speed of task performance also may decrease in these individuals/viewing condition, reflecting increased caution being exercised to avoid errors. Second, the functional depth vision of uniocular participants may be superior to monocular viewing of controls owing to such tasks being performed habitually by the uniocular group using monocular depth cues while it is a forced and unnatural behavior for the group with normal binocular vision. Third, the uniocular advantage may be more evident in individuals who are chronically uniocular from young ages, compared to acute and older cases, because of the visual system being overall more moldable^17^^,^^18^ and having greater habituation time in the former than latter cohorts.^19^^,^^20^ Fourth, the uniocular individuals may move their heads more than controls, as observed by Marotta et al.,^21^ facilitating the use of motion depth cues as a means of optimizing task performance; restricting these head movements will consequently hamper the performance.

## Methods

### Participants

The study was conducted at the L V Prasad Eye Institute (LVPEI), Hyderabad, India between September 2022, and April 2023. The study adhered to the tenets of the Declaration of Helsinki and was approved by the Institutional Review Board of LVPEI and the Optometry Proportionate Review Committee of City, University of London, UK. All adults participated in the study after signing a written informed consent form. Verbal consent was obtained from children <18 years of age while their parents/legal guardians signed the written informed consent form on their behalf. The cases recruited for this study were patients ranging from 6 to 37 years of age who were either congenitally uniocular, underwent enucleation/evisceration because of retinoblastoma, or suffered unilateral vision loss after trauma (Table 1). Excluded from this study were cases with an ambiguous history of vision loss, progressive loss of vision leading up to blindness in one eye, visual field loss, anomalous eye movements, any ophthalmic dysfunction in their functional eye, any systemic condition that restricted body movement, visibly shaking hands, or an inability to follow instructions. Standard clinical management was followed for all cases, with no influence of the study protocol on their clinical care. Age-similar, binocular controls were recruited from the institute's staff and student pool and their acquaintances, and from among the associates of patients visiting the institute. All participants had best-corrected, high-contrast, monocular near acuity of N6 or better at 40 cm viewing distance in both eyes (for binocular controls) or in their functional eye (for uniocular cases). Controls also had binocular stereoacuity of 50 arcsec or better at 40 cm viewing distance on the Titmus-fly test. None of the participants needed corrective lenses for near viewing. The sample size was calculated from the mean ± 1 SD of the just-noticeable difference in depth between two vertical rods reported by Gonzalez et al.^12^ To determine a difference in just-noticeable difference of 4 ± 3 mm for binocular viewing and 18 ± 18 mm for uniocular viewing with a study power of 80% and Type I error of 5%, the G*Power software (3.1.9.4 version, Franz Faul, Universität Kiel, Germany) estimated the necessary sample size to be 24 participants each in controls and cases.

**Table 1.** Demographic Details of Controls and Cases and the Cause of Uniocularity in Cases That Participated in the Study

	Age (Yrs)	Gender (M:F)	Age at Uniocularity (Yrs)	Duration of Uniocularity (Yrs)	Reasons
Cases (n = 45)					
Children (n = 16)	12 (9.8–15.3)	7:9	3.3 (1.2–4.1)	10.0 (6.4–11.3)	Retinoblastoma (n = 11); Trauma (n = 4); Anophthalmos (n = 1)
Adult (n = 29)	29 (23–31)	22:7	6.40 (3.4–16.8)	17.8 (9.7–25.9)	Retinoblastoma (n = 8); Trauma (n = 18); Microphthalmos (n = 1); Contracted socket (n = 1); Rhino-cerebral mucormycosis (n = 1)
Controls (n = 45)					
Children (n = 15)	10 (8.5–12)	11: 4	NA	NA	NA
Adult (n = 30)	26.5 (24.5–30)	11: 19	NA	NA	NA

Chronological age and the age and duration of uniocularity are reported as median (25^th^–75^th^ quartiles). NA, Not applicable.

### The Buzz-Wire Apparatus

Four buzz-wires were constructed from wires 0.10 cm in diameter. Three of these wires were 33.5 cm long and curved multiple times to provide for modulation in depth along the antero-posterior direction (see Fig. 1A for an example). The fourth wire was 10 cm long and remained unbent (Fig. 1B). Unlike Read et al.,^14^ the buzz-wires used in the present study did not have any vertical modulation; the bending was parallel to the tabletop. The insulated edges were clamped onto vertical posts separated by 11 cm. The vertical posts were fixed to a horizontal base that was, in turn, placed on a larger horizontal surface covered with white matte-finished paper. The wire pattern was mounted parallel to the horizontal base, thus resulting in continuous modulation in depth from one end of the vertical post to the other end (free-fuse the stereo pair in Fig. 1C to experience the depth structure). A 1-cm diameter and 0.3-cm thick metal hoop passed around the wire and was connected to a buzzer to deliver an audible sound each time the hoop contacted the wire (Fig. 1A). The stalk of the hoop was 9-cm long and was moved by hand along the length of the wire during each trial. The end of the buzz-wire was insulated so that the hoop could rest silently before and after the task. The entire buzz-wire apparatus, including the participant's face and part of the experimental surrounding, was video recorded using the standard front-camera setting of a cellular phone with an Android operating system (Redmi Note 5 Pro; Xiaomi, Beijing, China). The position of the mobile phone was fixed to a custom-built clamp at a 30-cm distance from the buzz-wire to ensure stability of the video recording. At this distance, the video recording subtended a viewing angle of 42° × 55° at the camera aperture of the mobile phone.

### The Buzz-Wire Task

The overall buzz-wire task is described in the introduction section. Participants were positioned 30 cm away from the buzz-wire at a mean (minimum to maximum range) elevation angle of 45° (36°–53°, depending on the height they sat in front of the apparatus) (Fig. 1A). For optimal overall engagement of the participants, the buzz-wire task was described as a “game” with the aim to make fewer errors, and the following instructions were given at the beginning of the game, verbatim in English or in the participant's local language:

This is a game in which the idea is to move this hoop along to the end of the wire without touching it. (1) Look at the camera without moving for 5 sec, during which I will give a verbal countdown and say “start”, upon which you will start the game. (2) Your task is to pass the hoop from one end to the other without touching the wire. (3) In case the hoop touches the wire, you will hear the buzzer ring. When you hear the buzzer, stop your movement, and make the buzzing stop by centering the wire within the circular hoop. (4) Once the buzzing stops, proceed forward until you reach the other end. (5) Make sure the hoop is held upright throughout the game.

No explicit instructions were provided on the speed with which the participant needed to perform the task or whether they could move their head while performing the task. These instructions were reiterated at the beginning of each experimental session. There were no prior practice sessions given to the participants.

All participants participated in the four versions of the wire pattern in random order. Controls performed the tasks under binocular and monocular viewing conditions, in random order, whereas cases performed the tasks only under uniocular viewing conditions. For monocular viewing of controls, one eye was randomly chosen to be occluded using a pirate patch. Additionally, the tasks were also performed with the participant's head free to move or with their head restricted using a chin and forehead rest. These too were performed in random order. The direction of movement of the hoop through the wire pattern (i.e., from the left end to the right end of the wire or vice versa) was determined randomly at the beginning of each session. In total, controls repeated the task 16 times (4 wire patterns × 2 viewing conditions × 2 head movement conditions = 16 repetitions) whereas cases repeated the task eight times (4 wire patterns × 1 viewing condition × 2 head movement conditions = 8 repetitions). Each task took approximately 40 seconds to complete, after which participants were given approximately one minute of break before the start of the next task to avoid fatigue and boredom. Once it was ensured that the participant was looking straight at the camera clamped in front of the buzz-wire (Fig. 1A), the examiner pressed the recording button on the phone. Performance on each buzz-wire of a participant was recorded separately for offline analysis. The examiner inspected every video for instances where the participant dragged the hoop along the wire. This resulted in the removal of nine trials from the binocular controls and three trials from the uniocular cases.

### Determination of the Outcome Variables in the Buzz-Wire Task

The videos were first cropped from the beginning of the task to the time the hoop entered the insulated portion of the other end of the wire. They were then converted to waveform audio file (.wav) format using custom-written software code in Python (3.10 Version, Centrum voor Wiskundeen Informatica, Amsterdam, The Netherlands) for analysis of buzzes using the open-source Audacity software (3.2.1 version, Audio.com, Boston, MA). Each audio file was plotted as a spectrogram that represented the signal strength in different frequency bands over time (Fig. 1D). The spectrogram was bandpass filtered to a frequency range of 4.0 to 4.1 kHz, and intensities within this frequency range were cut off at −30 db to effectively differentiate buzzes from the background noise (Fig. 1D). The task-completion time (i.e., the time between the verbal utterance of the word “Start” by the examiner to the end of the audio file), total number of buzzes and their time stamps corresponding to the onset and termination of each buzz were then saved for further analysis. From these, the error rate was calculated by dividing the number of errors made in the trial by the task completion time (errors/sec) and the speed was calculated by dividing the total length of the wire by the difference between task completion time and total error duration (cm/sec).

### Determination of the Location of the Error in the Buzz-Wire Task

The buzz-wire apparatuses with depth modulations contained locations of diverse degrees of difficulty that may have contributed unequally to the errors made during the task. For instance, the curved wire locations required the participants to veridically estimate both the slant and diastereopsis separation to avoid contact with the hoop and this would present a greater navigational challenge, relative to locations that are not curved (Figs. 1E–G). The curved locations may thus result in a greater number of errors, compared to the non-curved regions and would be greater for monocular and uniocular viewing than for binocular viewing. These hypotheses were tested by selecting a subset of frames from each of the buzz-wire videos where an error had occurred and judging the location of the hoop to be (1) at a straight portion of the wire or (2) at a transition zone from straight to the curved portion of the wire, or vice versa, or (3) at a curved portion of the wire (Figs. 1E–G). A total of 390 frames from videos of binocular, monocular, and uniocular viewing (130 frames for each viewing condition) were chosen at random across participants and the three buzz-wire apparatuses for this analysis. Each of these frames was shown in random order to three examiners who were naïve to the experiment objectives, and they were asked to make a forced-choice psychophysical judgement containing the three aforementioned alternatives. The identity of the participant and the viewing condition were masked by placing a black box over the participant's face to avoid examiner bias. No time limit was imposed on the examiners to decide on each presentation. The responses of the three examiners were tabulated for each video frame and the mode of the response choices was taken as the final location of the hoop along the wire. For instances where the response choices of the three examiners disagreed with each other, the decision of a fourth naïve examiner was sought and included in the calculation of the response choice mode.

### Analysis of Head Movements

All videos under the free head condition with depth-modulated buzz-wires were analyzed using the open-source software, OpenFace.^22^ This software detected and tracked facial landmarks that enabled tracking of the head's translational movements in the horizontal, vertical, and antero-posterior directions as well as its rotational movements about the yaw, pitch, and roll axes (Figs. 1H–J). Localization of facial landmarks failed in 37% of these videos because of obstruction of the face by the hand (see Fig. 1J for an example) or a sudden, unexpected change in the video quality. These videos were removed from the analysis. What remained were binocular trials from 40 cases (children: 14; male: 19) and monocular trials from 31 cases (children: 12; male: 21) of the 46 controls, and 32 trials from 45 uniocular cases (children: 12; male: 21). Once the participant was instructed to start the task, a few of the participants were noted to adopt a preferential head tilt to get comfortable before starting the task. The range of translational and rotational head movement was calculated as the difference between the maximum and minimum head position and frontal orientation along the horizontal, vertical, and antero-posterior axes during the task. The head movement velocity was obtained by dividing the total magnitude of horizontal head translation by the task completion time.

The magnitude of the head's horizontal translation was used to calculate the equivalent disparity generated from motion parallax for computing the depth between the wire and one side of the hoop edge during the buzz-wire task (Equation 1). This calculation was performed only for the monocular viewing of controls and for uniocular participants, where motion parallax becomes a primary cue for computing depth, in the absence of binocular retinal disparity. Since the head velocity of the participants was quite slow, it was assumed that the motion parallax signal was derived primarily from the relative retinal image velocities between the wire and the hoop edge, without any influence of head velocity.^23^^,^^24^ Equivalent retinal disparities from binocular viewing of controls were calculated using Equation 2.^3^ For both the equations, the distance between the wire and one side of the hoop edge was constant (Δ*D* = 4 mm). The anteroposterior distance from the participant to the buzz-wire apparatus (*D*) and the magnitude of horizontal head translation (Δ*H*) were obtained from the head motion data obtained earlier. The interocular distance (*IOD*) for calculating binocular retinal disparity was obtained from age-appropriate values described in MacLachlan and Howland.^25^

(1) $$\begin{array}{c} \delta_{\mathrm{ed}}\;=\frac{\Delta H\;\times \;\Delta D}{D^{2}}\times \;\frac{60\;\times \;180}{\pi } \end{array}$$

(2) $$\begin{array}{c} \delta_{\mathrm{rd}}\;=\frac{IOD\;\times \;\Delta D}{D^{2}}\times \;\frac{60\;\times \;180}{\pi } \end{array}$$

where, δ_ed_ = Equivalent disparity from head movements (arcmin); δ_rd_ = Binocular retinal disparity (arcmin); Δ*H* = Magnitude of horizontal head translation (mm); Δ*D* = Distance between the wire and the edge of the hoop (mm); *D* = Anteroposterior distance between participant and the buzz-wire apparatus (mm); *IOD* = Interocular distance (mm).

### Statistical Analysis

Statistical analysis was performed using IBM SPSS Statistics (Version 21; Armonk, NY) and Matlab (R2016a). The three repetitions of the buzz-wire task exhibited good test-retest repeatability and did not show any short-term practice effects (Appendix 1). The data from the three repetitions were therefore averaged for further analyses. The Shapiro Wilk test revealed that the dependent factors of error rate, speed, and the magnitude of translational and rotational head motion were not normally distributed. Parametric tests are more powerful at revealing data trends in normally distributed data than in non-normally distributed data. Thus, all dependent variables were transformed by taking their square root to achieve normality, thereby properly conditioning the analyses for the use of parametric statistics (note, however, that all the figures except Figures 3 and 5 are constructed on the raw untransformed data for visualization purposes). A five-factor repeated measures multiple analysis of variance (RM-MANOVA) was performed on the data of controls, to investigate the between-subject factors of age (children vs. adult) and gender (male vs. female), and the within-subject factors of viewing condition (binocular vs. monocular), buzz-wire pattern (curved vs. straight) and head position (free vs. fixed) on the dependent variables of error rate and speed. Age was not considered as a covariate as it showed a significant interaction with other independent variables.^26^ Age was instead treated as a between-subject independent variable by categorizing participants into children (≤18 years of age) and adults. This age categorization was also confirmed by regressing the square root of error rate against age using a bilinear model. The kink point in the bilinear fit occurred at 16.6, 17.5 and 20.0 years for binocular, monocular and uniocular conditions, respectively *(data not shown)*, consistent with the formal 18-year age-point for the transition to adulthood. A separate four-factor MANOVA was performed on the data of uniocular cases to investigate the effect of between-subject factors age and gender and within-subject factors buzz-wire pattern and head position on the same two dependent variables.

In addition, a forward stepwise linear regression analysis was used to identify the possible predictors of the square root-transformed error rate in children and adults from amongst the candidate variables of age and the duration of uniocularity.^26,27^ At each step of the regression analysis, the variables were added based on their *P* values, with a threshold of *P* ≤ 0.05 as a limit on the total number of variables to be included in the final model. A backward stepwise linear regression analysis revealed the same results as the forward analysis and, hence, not reported here separately. This analysis was not performed on speed because there was no impact of age on this dependent variable. Also, the age of uniocularity was not included in the analysis as this was simply the difference between the participant's age and their duration of uniocularity.

The effect of the magnitude of head movements among the three viewing conditions and two age groups was analyzed using a two-factor MANOVA analysis. The six degrees of head movements (three translations and three rotations) were considered as dependent variables. Viewing condition (binocular vs. monocular vs. uniocular) and age groups (children vs. adults) were considered as a between-subject factor. *P* value < 0.05 was considered statistically significant in all analyses.

## Results

Table 1 shows the demographic details of the participants along with the reason for uniocularity in cases. The participants’ age (*P* = 0.76) and gender (*P* = 0.49) were not significantly different between cohorts.

### Cohort-Level Task Performance

**Figure 2.**
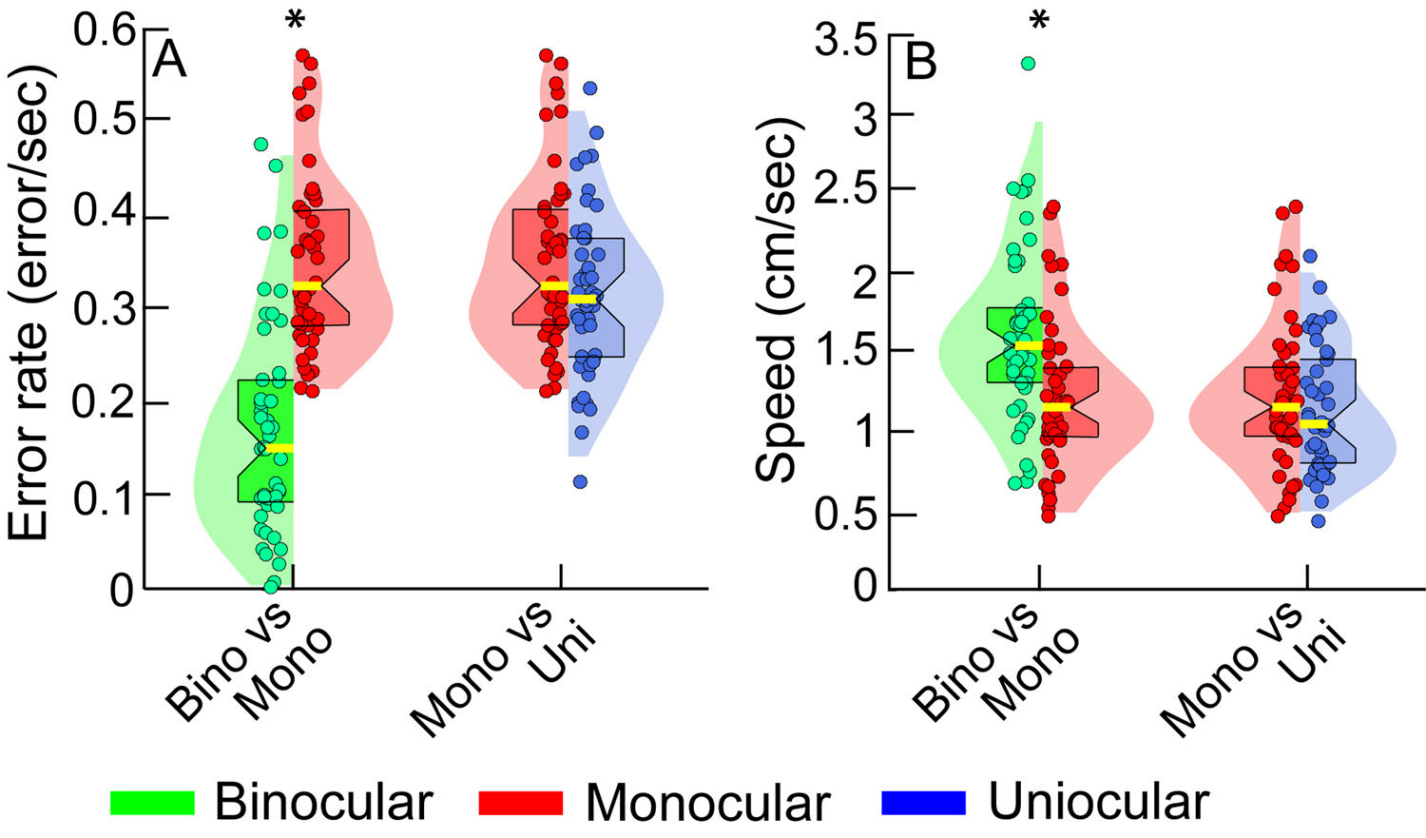
Violin plot pairs showing the distribution of the error rate (**A**) and speed (**B**) for the binocular and monocular viewing of controls and for uniocular cases while they performed the buzz-wire tasks with depth modulation under head-free viewing conditions. Each violin plot is constructed with a kernel density that was calculated by taking the maximum and minimum data range for each outcome variable in a given cohort and dividing it into ten equal bins. Superimposed within the violin plots are box and whisker plots, with the central yellow solid line within each plot, indicating the median value, the notch of the box indicating the 95% confidence interval of the median and the edges of the box indicating the 25th and 75th percentile. The violin plot is truncated at the 1st and 99th percentile. The *circles*, with random jittering along the abscissa indicates the individual subjects’ error rate, averaged over the three trials with the depth modulated buzz-wire and under head free condition. The *asterisk* indicates statistical significance at *P* < 0.05.

Figure 2 shows a combination of violin and box-and-whisker plots for the two outcome measures obtained from all controls and cases while they performed the buzz-wire task with depth modulation under head-free conditions. The median (25th–75th quartiles) error rate under binocular viewing condition of controls [0.15 errors/sec (0.09–0.22 errors/sec); i.e., an error every 6.67 seconds] was smaller than their monocular viewing [0.33 errors/sec (0.28–0.41 errors/sec); i.e., an error every 3.03 seconds] and that of the uniocular subjects [0.31 errors/sec (0.25–0.38 errors/sec); i.e., an error every 3.23 seconds] (Fig. 2A). Controls moved significantly faster under binocular conditions [1.55 cm/sec (1.31–1.87 cm/sec)], relative to their monocular viewing [1.16 cm/sec (0.97–1.44 cm/sec)] and relative to the uniocular cohort [1.05 cm/sec (0.81–1.46 cm/sec)] (Fig. 2B).

### Multivariate Analysis of Task Performance in Controls

**Figure 3.**
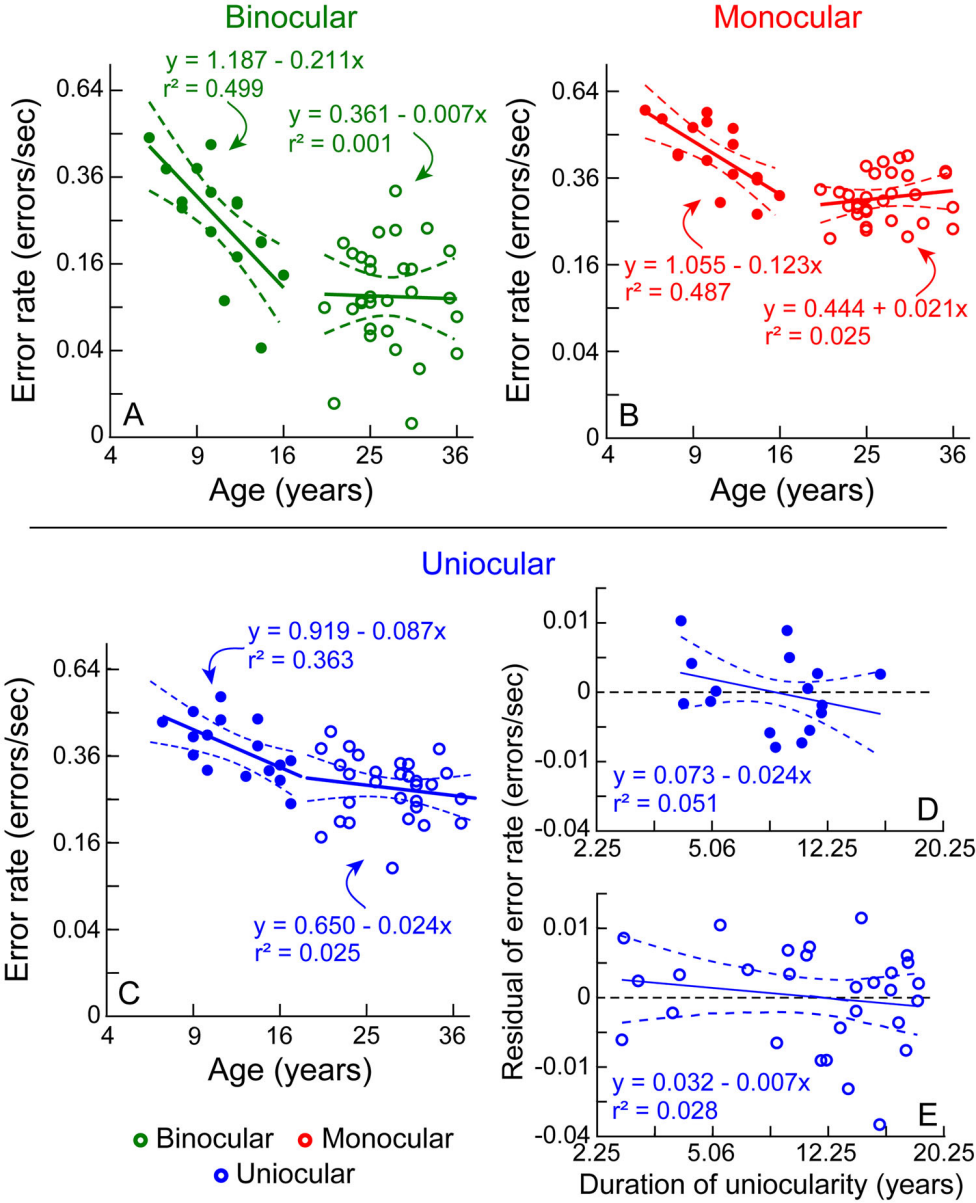
(**A–C**) Scatter diagrams of the error rate plotted as a function of the square root of the participant's age under binocular (**A**), monocular (**B**), and uniocular (**C**) viewing conditions. (**D, E**) Partial residual plots for children (**D**) and adults (**E**) demonstrating the impact of the duration of uniocularity on the error rate after adjusting for the effect of the participant's age, as shown in panel **C**. The solid and curved lines in each panel indicate the best-fit linear regression equation and its ±95% confidence interval obtained for the data of children (*closed symbols*) and adults (*open symbols*), separately. The abscissa and ordinate (**A–C**) are relabeled for the untransformed age and duration of uniocularity for ease of interpretation. Similarly, the abscissa and ordinate of **D** and **E** are relabeled for the untransformed duration of uniocularity and residuals of the error rate for ease of interpretation.

**Figure 4.**
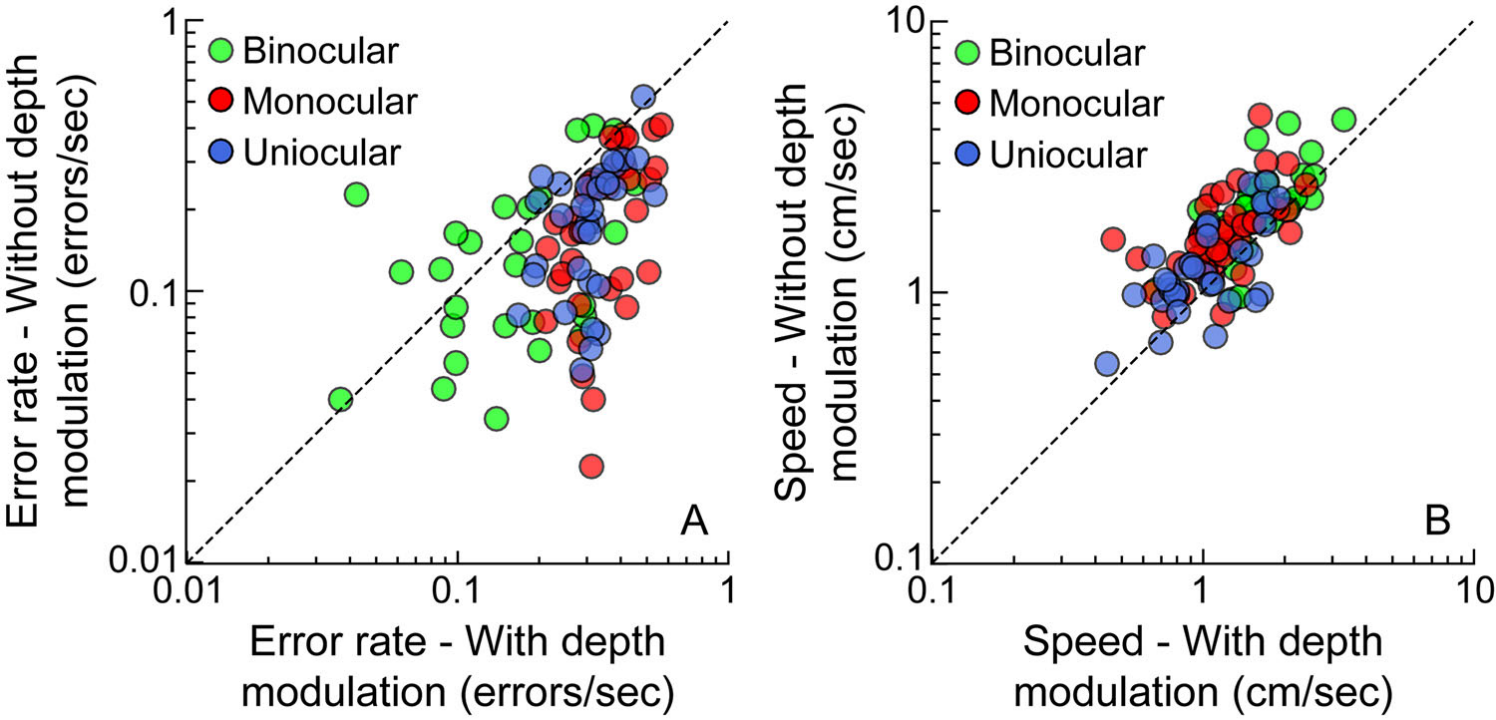
The error rate (**A**) and speed (**B**) obtained for buzz-wires with depth modulation plotted against those without depth modulation in controls and cases in the free head condition. The *diagonal line* in each panel represents the line of equal performance.

The five-factor RM-MANOVA between binocular and monocular viewing of controls revealed significant main effects of age, viewing condition and buzz-wire pattern on the combined dependent variables (Table 2). Among these factors, age group was statistically significant for the error rate but not for the speed (Table 2). The relationship between the square root of the error rate and the square root of age for binocular and monocular viewing conditions is shown in Figures 3A and B, respectively. The data of children showed a decrease in error rate at the rate of 0.03 errors/sec and 0.002 errors/sec per unit increase in age under binocular and monocular viewing conditions, respectively. The equivalent data for adults showed no significant change with age, but the y-intercepts showed an overall lower error rate under binocular (0.35 errors/sec) than monocular (0.50 errors/sec) viewing conditions (Figs. 3A, 3B). The interaction between viewing condition and buzz-wire pattern was also significant (Table 2, Fig. 4).

**Table 2.** The Results of Five-Way RM-MANOVA Comparing the Binocular and Monocular Task Performance of Controls

		F	*P* Value	Partial ƞ^2^
Multivariate Tests				
Age group		13.7	**<0.001**	0.45
Gender		2.3	0.12	0.12
Viewing condition		49.3	**<0.001**	0.74
Buzz-wire pattern		35.6	**<0.001**	0.68
Head position		1.00	0.37	0.06
Viewing condition × Age group		3.1	0.06	0.16
Viewing condition × Buzz-wire pattern		7.1	**0.003**	0.29
Viewing condition × Head position		2.2	0.13	0.11
Buzz-wire pattern × Age group		0.4	0.69	0.02
Head position × Age group		0.1	0.95	0.00
	**Error Rate (Errors/sec)**		**Speed (cm/sec)**	
	**Mean ± SE**	***P* Value**	**Mean ± SE**	***P* Value**

Univariate Tests				
Age group		**<0.001**		0.56
Children	0.50 ± 0.02		1.27 ± 0.05	
Adults	0.37 ± 0.02		1.23 ± 0.04	
Viewing condition		**<0.001**		**<0.001**
Binocular	0.36 ± 0.02		1.31 ± 0.04	
Monocular	0.51 ± 0.01		1.19 ± 0.04	
Buzz-wire pattern		**<0.001**		**<0.001**
With modulation	0.51 ± 0.01		1.18 ± 0.03	
Without modulation	0.37 ± 0.02		1.33 ± 0.04	

Multivariate test results are shown for the main effects and for interaction between relevant independent variable pairs. Univariate test results are shown for variables that were significant in the multivariate test. Relationships with *P* < 0.05 appear in bold. The mean ± standard error (SE) are the square-root-transformed values. The means need to be squared for comparison with the data shown in the figures.

**Figure 5.**
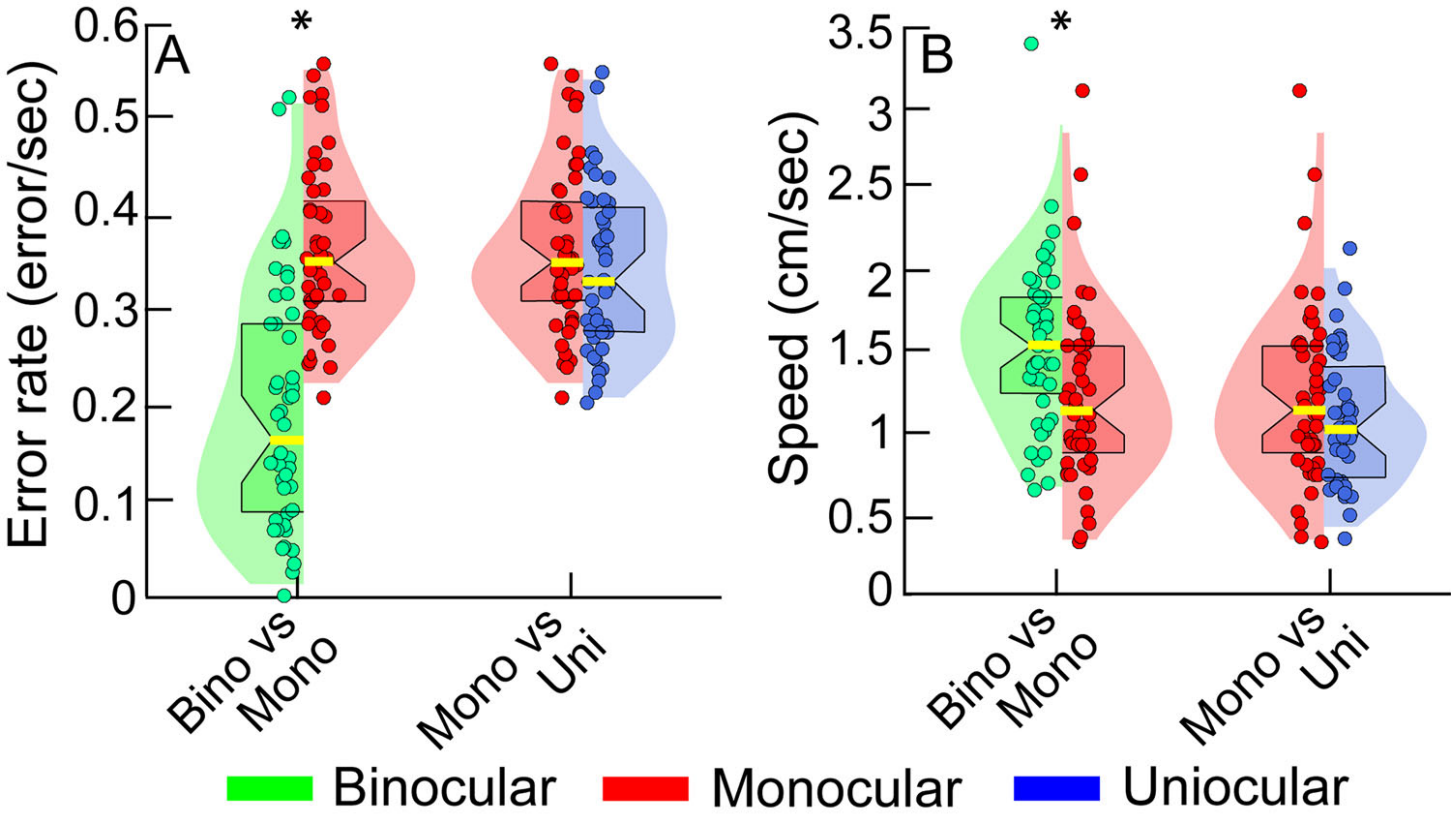
Similar to Figure 2 but buzz-wire performance for the head-restricted viewing condition.

The error rate was higher, and the speed was lower for the buzz-wire with depth modulation than without depth modulation, more so under monocular than binocular viewing conditions (Fig. 4), reflecting the significant interaction between the viewing condition and buzz-wire pattern in Table 2. Unlike the buzz-wire pattern, the head-free and restricted conditions did not have any impact on the error rates or on speed among controls viewing under binocular and monocular condition (Table 2 and Fig. 5). The pattern of the violin plots in Figure 5 for the head-restricted viewing was very similar to that of the head-free viewing condition in Figure 2.

### Multivariate Analysis of Task Performance in the Uniocular Cases

The four-factor MANOVA revealed a significant effect of age on the error rate but not on the speed (Table 3, Fig. 3C). As was the case in controls, Figure 3C shows that there was a reduction in the error rate at the rate of 0.013 errors/sec per unit increase in age for children but no change in the error rate with age in adults. The forward stepwise regression analysis revealed that only age was a statistically significant predictor of the error rate in children (Table 4, Fig. 3D). The addition of the duration of uniocularity increased the r^2^ estimate of the regression model by 9% in the data of children, but this increase was not statistically significant (Table 4, Fig. 3D). Neither age nor the duration of uniocularity were found to be statistically significant predictors of error rate in adults (Table 4, Fig. 3E). The MANOVA analysis also revealed a significant main effect of the buzz-wire pattern on the error rate and speed (Table 3, Fig. 3).

**Table 3.** The Results of the Four-Way Repeated Measures MANOVA Comparing the Monocular Task Performance of Controls With Uniocular Performance of Cases

		F	*P* Value	Partial ƞ^2^
Multivariate Tests				
Gender		0.08	0.92	0.01
Age group		10.17	**0.001**	0.43
Buzz-wire pattern		73.51	**<0.001**	0.85
Head position		3.16	0.06	0.19
Buzz-wire pattern × Age group		7.35	**0.003**	0.35
Head position × Age group		0.23	0.80	0.02
	**Error Rate (Errors/sec)**		**Speed (cm/sec)**	
	**Mean ± SE**	***P* Value**	**Mean ± Se**	***P* Value**

Univariate Tests				
Age group		**<0.001**		0.08
Children	0.56 ± 0.02		1.15 ± 0.05	
Adults	0.47 ± 0.01		1.03 ± 0.04	
Buzz-wire pattern		**<0.001**		**<0.001**
With modulation	0.58 ± 0.01		1.04 ± 0.04	
Without modulation	0.45 ± 0.02		1.15± 0.03	

^*^All other details are the same as in Table 2.

**Table 4.** The Results of Step-Wise Multiple Regression Investigating the Relationship Between the Error Rate With Uniocular Participant's Age Alone and on Adding the Duration of Uniocularity Into the Regression Model

		Change Statistics		
Model	*r* ^2^	*r* ^2^ Change	F Change	Sig. F Change
Children				
Age Only	0.36	0.36	7.93	**0.01**
Age + Duration of Uniocularity	0.46	0.09	2.27	0.16
Adult				
Age Only	0.03	0.03	0.69	0.41
Age + Duration of Uniocularity	0.05	0.03	0.74	0.40

Relationships with significance *P* < 0.05 appear in bold.

The uniocular cases performing the buzz-wire task with depth modulation resulted in higher error rate and lower speeds than those without depth modulation (Table 3, Fig. 4). As for the controls, head position did not have any impact on the error rate and speed for the uniocular cases (Table 3, Fig. 5).

### Analysis of the Location of the Error in the Buzz-Wire Task

**Figure 6.**
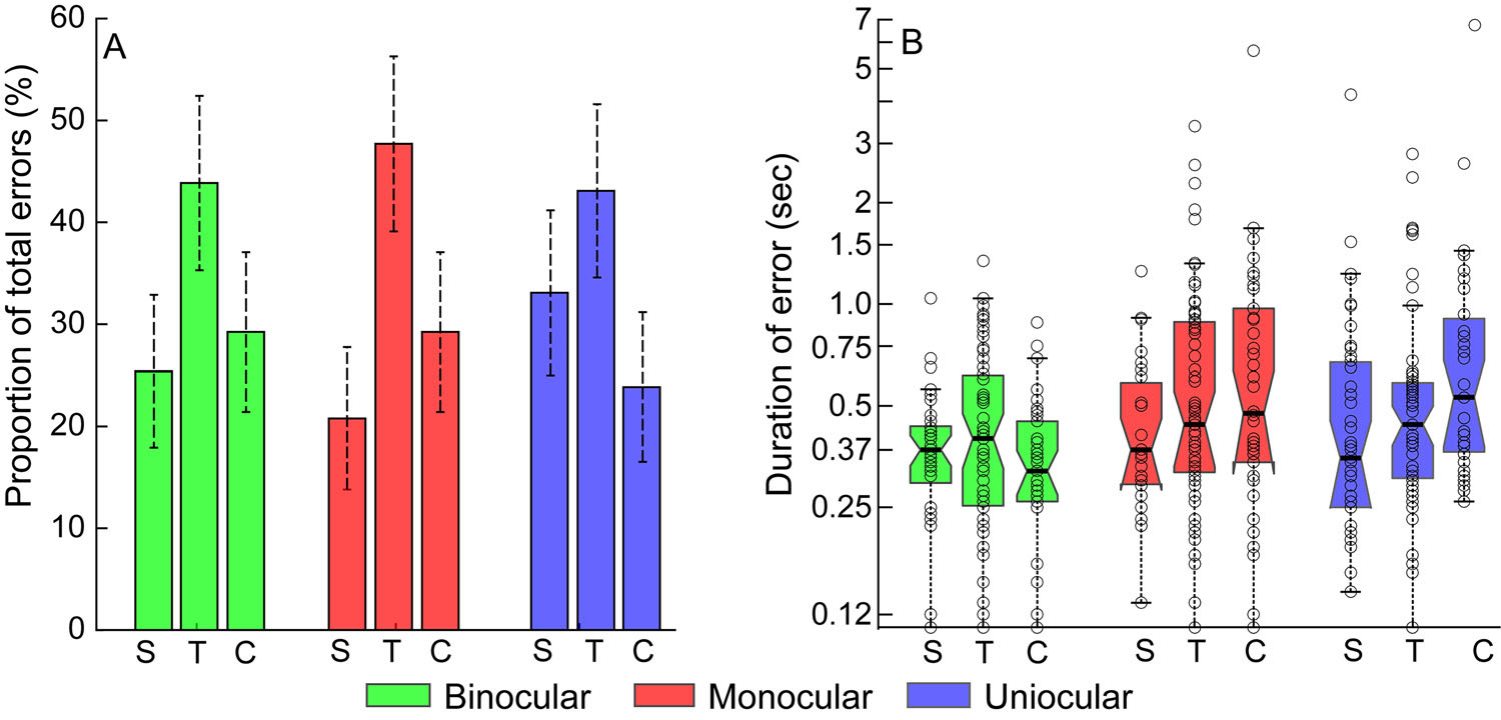
(**A**) Histograms of the proportion of errors made at the straight (S), transition (T), and curved (C) regions of the buzz-wire across the randomly selected error frames analyzed under binocular (*green*) and monocular (*red*) viewing conditions of controls and uniocular viewing condition of cases (*blue*). *Error bars* indicate the upper and lower (95%) confidence interval of each proportion shown in this panel. (**B**) Box and whisker plots of the duration of errors made by controls under binocular and monocular viewing conditions and by uniocular participants in each of the locations of the wire. The *middle black horizontal line* in each box and whisker plot indicates the median value, the lower and upper horizontal lines indicate the 25th and 75th quartiles and the dotted vertical lines indicate the 1st and 99th percentile of the data distribution. The *open circles* represent the data of individual participants.

Figure 6A shows the histogram of the proportion of errors made by controls under binocular and monocular viewing and by uniocular participants in the straight, curved, and transition portions of the buzz-wire. Of the 390 video frames with errors that were analyzed, close to half the frames showed errors being made in the transition portion of the wire track [Binocular: 45.4% (95% confidence interval {CI} of proportion: 36.8–53.9%); Monocular: 50.0% (41.4%–58.6%); Uniocular: 43.1% (34.6–51.6%)]. The remaining errors were approximately equally distributed between the straight [Binocular: 25.4% (17.9–32.9%); Monocular: 20.8% (13.8–27.7%); Uniocular: 33.1% (25.0–41.2%)] and curved [Binocular: 29.2% (21.4–37.1%); Monocular: 29.2% (21.4–37.1%); Uniocular: 23.9% (16.5–31.2%)] portions of the wire track. A χ^2^ test did not reveal any association between the location of errors and the viewing condition [χ^2^(4) = 5.42; *P* = 0.25]. Unlike the error proportions, the duration of the errors did not show any significant difference across the three regions of the buzz-wire [χ^2^(2) ≥ 2.20, *P* ≥ 0.33, for all] (Fig. 6B). As expected, the transition zone in the wire-track resulted in the maximum number of errors during the task, relative to the other two locations.

### Analysis of Head Movements

In general, as stated in the Methods, the study participants made two kinds of head movements during the task (see Appendix 2 for face tracking videos of representative study participants). At the beginning of the task, almost all participants moved their heads to a “preferred” position, which was in the direction opposite of their dominant hand. From this position, some participants moved their heads monotonically in the same direction of the hoop motion during the buzz-wire task, whereas others made to-and-fro head movements during the buzz-wire task (Appendix 2). Participants sometimes also made vertical translational movements of the head, fore- and aft-movement of the head in the anteroposterior direction and all three directions of rotational head movements during the task (Appendix 2). These movements were rather idiosyncratic and did not correspond to when an error was made during the task.

**Figure 7.**
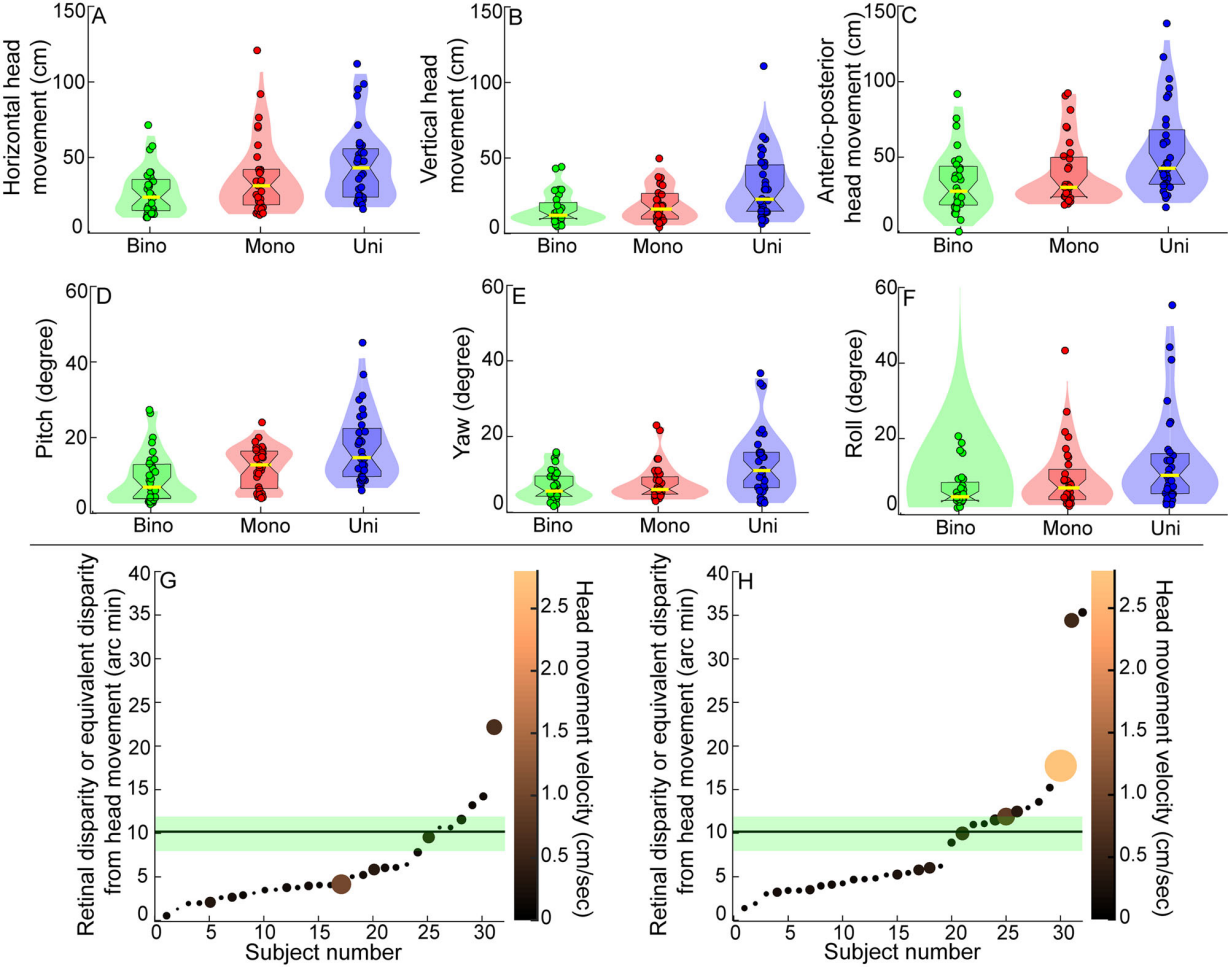
Violin plots showing the distribution of the translational [horizontal (**A**), vertical (**B**) and anteroposterior (**C**)] and rotational [pitch (**D**), yaw (**E**) and roll (**F**)] head movements for the binocular and monocular viewing of controls and for uniocular cases while they performed the buzz-wire tasks with depth modulation under head-free viewing conditions. All other details are similar to Figure 2. Bubble plots showing the disparity available for depth computations in the buzz-wire task with depth modulations from horizontal head translation in the monocular viewing of controls (**G**) and in uniocular cases (**H**). In both panels, the participants are arranged in ascending order of the disparity available to each participant in the cohort. Lighter-colored and larger-sized bubbles indicate a larger velocity of head motion. The *horizontal line* with the green-shaded area in each panel denotes the median with 25th and 75th quartile of binocular retinal disparity available for depth calculations under binocular viewing conditions.

Figures 7A through 7F shows the magnitude of head movements made by the participants from their preferred position under binocular, monocular and uniocular conditions. The multivariate analysis performed on these data revealed a significant impact of viewing condition (*P* = 0.003) and age (*P* = 0.04) on the magnitude of head movements, but with no interaction between these factors (*P* = 0.12) (Table 5). Univariate comparison revealed significantly larger translational movements and head rotations (except roll head movement) in the uniocular conditions, compared to the binocular condition (*P* ≤ 0.01, for all). Compared to monocular viewing condition, the uniocular participants made larger vertical, anteroposterior and yaw head movements (*P* ≤ 0.03, for all). There was no difference in these head movements between binocular vs. monocular (*P* ≥ 0.24, for all). Univariate comparison revealed children made statistically significantly larger translational and rotational head movement (except pitch head movement), compared to adults (*P* ≤ 0.02, for all). The median head speed of the monocular controls was 0.15 cm/sec (0.08–0.21 cm/sec) (Fig. 7A) and for uniocular cases was 0.16 cm/sec (0.10–0.28 cm/sec) (*P* = 0.7; Fig. 7G), with the maximum head speed of 2.75 cm/sec generated by a uniocular participant (Fig. 7H).

**Table 5.** Results of the Two-Way MANOVA Comparing the Head Movements Made by Controls Under Binocular and Monocular Conditions, and by Uniocular Participants

			F	*P* Value	Partial ƞ^2^
Multivariate Tests					
Viewing			2.6	**0.003**	0.95
Age group			2.38	**0.04**	0.13
Viewing × Age group			1.53	0.12	0.09
	**Viewing Condition**			**Age**	
**Head Movements**	**Bino**	**Mono**	**Uni**	**Children**	**Adult**

Univariate tests					
Horizontal	5.38 ± 0.27	6.00 ± 0.32	6.66 ± 0.31^*^	6.44 ± 0.28^‡^	5.59 ± 0.21
Vertical	3.96 ± 0.22	4.27 ± 0.26	5.50 ± 0.24^*^^,^**^†^**	4.96 ± 0.22^‡^	4.20 ± 0.16
Anteroposterior	5.72 ± 0.28	6.05 ± 0.33	7.26 ± 0.31^*^^,^**^†^**	6.81 ± 0.29^‡^	5.88 ± 0.21
Pitch	0.40 ± 0.02	0.46 ± 0.03	0.54 ± 0.03^*^	0.49 ± 0.02	0.45 ± 0.02
Yaw	0.35 ± 0.02	0.35 ± 0.02	0.46 ± 0.02^*^^,^**^†^**	0.43 ± 0.02^‡^	0.36 ± 0.02
Roll	0.37 ± 0.03	0.40 ± 0.04	0.48 ± 0.04	0.46 ± 0.03	0.38 ± 0.02

The mean ± standard error (SE) shown here are the square-root-transformed values, as described in the Methods section. Mean values need to be squared for comparison with the data shown in the figures.

* Statistically significant differences in head movements between binocular and uniocular conditions and between monocular and uniocular conditions.

† Statistically significant differences in head movements between binocular and uniocular conditions and between monocular and uniocular conditions.

‡ A significant difference between children and adults.

The median (25th–75th quartile) equivalent disparity from horizontal translation head movements was 4.08 arcmin (2.89–7.83 arcmin) for monocular controls (Fig. 7G) and 5.63 arcmin (3.97–11.82 arcmin) for the uniocular cases (Fig. 7H). The median retinal disparity for binocular controls was 10.18 arcmin (7.60–11.91 arcmin) (Figs. 7G, 7H). These values were significantly higher than the thresholds for detecting depth from motion parallax (∼1–1.3 arcmin^3^^,^^23^^,^^24^^,^^28^) and retinal disparity (clinically accepted stereo threshold = 0.67 arcmin, or 40 arcsec^8^) reported in the literature (monocular and binocular depth detection thresholds were not estimated in the present study cohort). Thus, even while suprathreshold levels of disparity from motion parallax were available to the participants in the absence of binocularity, they failed to impact the buzz-wire task performance, as observed from the similarity of the results in the head-free and head-restricted viewing conditions in Tables 2 and 3. As expected, the disparity variations derived from head motion were also not significantly correlated to the error rate in the buzz-wire task (*P* > 0.11, for all).

## Discussion

Given that the functional vision status of an individual is heavily task-dependent,^13^ it was important to choose a task that would provide as unbiased answers as possible for the questions raised in the present study. For the study, it was imperative that the functional depth task chosen for evaluation could demonstrate the expected worsening of task performance under monocular viewing, relative to binocular viewing.^11^^,^^14^^,^^15^^,^^21^^,^^29^^–^^31^ The present results affirmed this trend for the buzz-wire task by showing an increase in the error rate (by an average factor of 2.2) and a reduction in movement speed (by an average factor of 1.3) under monocular conditions, relative to binocular viewing by controls (Table 2, Fig. 2). These results are similar to the observations of Read et al.,^14^ who showed 3.5-fold increase in the number of errors and 1.3-fold increase in the task duration under monocular viewing, relative to binocular viewing. The data also compare well with the observations of Joy et al.^15^ and Piano and O'Connor^11^ who showed increases in the task-completion times under monocular relative to binocular viewing, on buzz-wire and bead-threading tasks, respectively. Neither of these studies reported the errors encountered during the task, however. Finally, it is important to point out that reduced speed under monocular viewing was not accompanied by any reduction in error rate. Indeed, like those in Read et al.^14^ the participants of the present study also made more errors under monocular viewing conditions, relative to their binocular viewing performance (Table 2, Fig. 2). In other words, there was no significant speed-accuracy trade-off in the buzz-wire performance.

### The Effect of Age on Performance

The primary effect in Tables 2 and 3 is the effect of age on performance − error rates were found to decrease with the age of our younger participants, falling to adult levels somewhere near 16 years of age (Figs. 3A–C). This age of “visuomotor maturity” may be compared with previous maturity trends described for a manual dexterity task involving adaptation/de-adaptation to purposely induced errors in a visuomotor task^20^ and for a visual function task involving detection of contrast-modulated flicker.^32^ The rate of adaptation/de-adaptation in the former task is relatively constant in individuals until their mid-twenties, after which they speed up until the late-forties before deteriorating again.^20^ This time span corresponds poorly with the maturity function described here for the buzz-wire visuomotor task (Fig. 3A–C). Instead, the present maturation curve corresponds much better with the age at which flicker sensitivity reaches adult levels (14–18 years), suggesting that the maturation of accuracy in visuomotor performance might be influenced by the contrast processing capabilities of the developing visual system.^32^ A causal relationship between the two, however, need to be established in the future. Whatever be the reason for the maturation trends, the results clearly showed that the binocular task accuracy improved at nearly twice the rate of the monocular and uniocular viewing (Figs. 3A–3C). This observation is in line with previous reports of binocular cues being weighted more than monocular cues for depth-related visuomotor tasks, relative to tasks involving the perception of depth^33^ and with the reports of binocular vision contributing to the training and maturation of the visuomotor system via the disparity processing in “action control” areas of the posterior parietal cortex.^34^^,^^35^ Having established task relevance and the age effect, the present study outcomes may be used to answer the questions raised in the introduction section about the functional-depth related task performance of individuals who have lived with uniocular enucleation over extended periods of time.

### Is the Task Performance of Uniocular Individuals Better Than Expectations From Chronological Maturity?

The present results fail to provide statistical evidence for an impact of the duration of uniocularity on task performance, beyond the age effect (Table 4, Figs. 3C, 3D). As observed in Figures 3B and 3C, there was no difference in the developmental trend of error rates between uniocular children compared to binocular children who were temporarily made monocular. Children constituted only 35.5% (16 out of 45) of the total uniocular cohort but their durations of uniocularity were also long in relation to their age (5–15.5 years) (Table 1). The acute effects of uniocularity over the first few months, which may have revealed the maximum impact of this factor on the error rates, thus could not be captured in the present dataset. Adults in this study did have a large range in the duration of uniocularity (2.4 months to 31.2 years) and even in this cohort, the duration of uniocularity failed to reveal any statistically significant impact on the error rates (Table 4, Fig. 7E). That there may be a positive influence of very acute durations of uniocularity (<2 months; the shortest duration of uniocularity in this study) on the error rates of visuomotor tasks like the one used here remains open for further investigation. These analyses were performed on a cross-sectional dataset that was not designed to parse out the relative impacts of age and duration of uniocularity on task performance. Future studies may address this limitation by recruiting children and adults with even shorter durations of uniocularity but bearing in mind the challenges of collecting data in such a cohort acutely following a traumatic medical experience. Despite these limitations, the forward stepwise linear regression on the children's data did show an approximately 6.7% improvement in the error rate with the duration of uniocularity after accounting for the age effect, albeit failing to reach the statistical significance (Table 4). Overall, this study presently rules out the influence of duration of uniocularity on depth-related visuomotor task performance, but leaves the possibility open for such an effect to become manifest in a dataset focused on individuals with more acute durations of uniocularity.

### Clinical Implications of the Results

That the duration of uniocularity may have only a modest influence on visuomotor task performance has important implications for the clinical management/rehabilitation of one-eyed individuals. Although some children are born without a fully developed eye (microphthalmos or anophthalmos; for the present cohort, see Table 1), others lose an eye at an early age because of retinoblastoma or trauma. Advanced retinoblastoma is typically managed by removing the entire eyeball from the orbit (enucleation), often to save the child's life.^36^ Based on the data from a tertiary eye care center, 95% of all enucleation procedures are performed on children, half of which are performed on eyes with tumors like retinoblastoma.^37^ Indeed, individuals whose eye was enucleated for retinoblastoma constituted the largest cohort of participants in the present study (Table 1). As expected, such a radical medical procedure has been reported to have significant psychological impact on the quality of life of these patients.^38^ Eye care practitioners often focus only on the anatomical health of the afflicted and fellow eyes of the uniocular patient during an eye examination (e.g., signs of recurrence of retinoblastoma tumor or infection in an enucleated socket), neglecting the functional ramifications of the loss of binocularity in their patients. The present study outcomes suggest that their depth-related functional vision is likely to remain deficient, irrespective of the duration for which they remain uniocular. Children who lose one eye may improve in their functional depth performance owing to general visuomotor maturation,^39^^,^^40^ but this is not readily attributable to them getting habituated to performing routine tasks with only one eye. This inference resonates well with instances of uniocular patients reporting difficulties in depth-related activities of daily living that may hamper their quality of life (e.g., boarding the stairs of a bus, fitting a bulb onto a bulb holder, or inserting the test strip into the slot of a blood-glucose monitoring device).^41^^–^^43^ Other aspects of vision that have a strong binocular influence, especially during the critical period of binocular vision development, may also show deficiency in these patients.^44^^–^^48^ Optokinetic nystagmus responses of unilaterally enucleated children, for instance, show more asymmetry than those of typically developing children.^44^^,^^45^ Ocular accommodation that is heavily dependent on binocular vergence input in the first decade of life also shows significant gain loss in one-eyed children, vis-à-vis, their binocular counterparts.^49^ All these issues must be considered by the eye care practitioner to offer appropriate counselling to one-eyed patients for optimizing their daily functioning.

**Figure A1.**
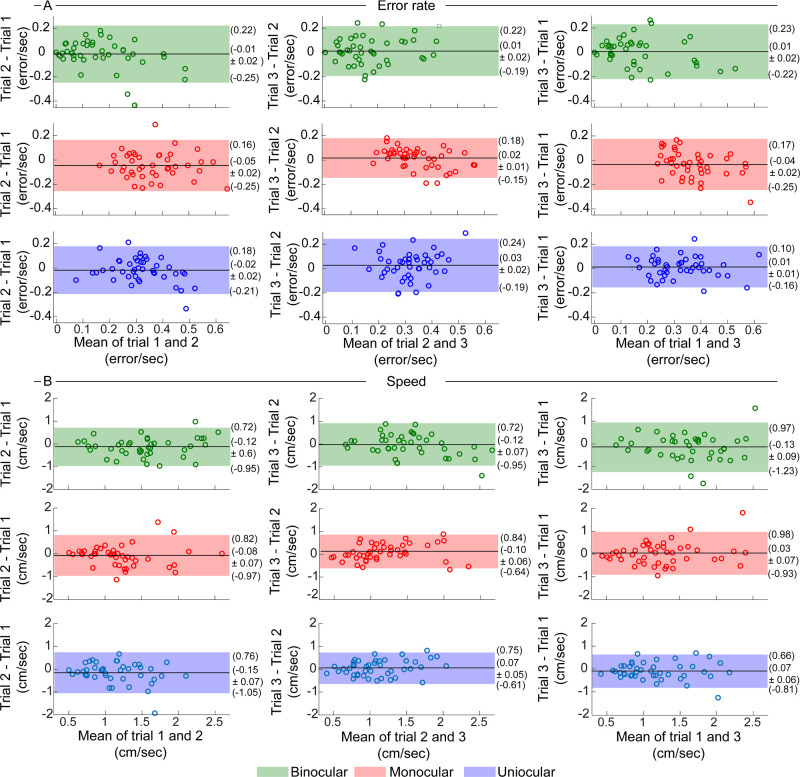
Bland-Altman plots for error rate (**A**) and speed (**B**) observed across the three trials of the buzz-wire task with depth modulation under head-free viewing for individual controls under binocular (*green*) and monocular (*red*) viewing conditions (*top panel*) and for the uniocular participants (*bottom panel*). The left columns compare data obtained from trials 1 and 2, the middle columns compare data obtained from trials 2 and 3 and the right columns compare data obtained from trials 1 and 3. In each panel, the solid black line represents the mean difference, with the value mean±1SD of the mean mentioned on the right side of the line. The upper and lower edge of the colored box indicates the upper and lower limits of agreement, respectively, with the specific value specified on the right of the edges.

Having said all this, there are documented reports of individuals who, despite losing an eye, participate in professional activities that require an acute sensation of stereo vision (e.g., piloting an aircraft,^50^ Formula one racing,^51^^,^^52^ heavy-duty truck driving,^53^ professional cricketing,^54^ film directors^55^.) This leaves open the possibility that their judgment of depth did get refined due to practice with the task at hand. While the present study did not show any learning effect over the three repeated trials (Figs. A1A, A1B), it was not designed to address the impact of practice on the buzz-wire task performance. Future studies may address this issue more systematically. Future studies may also investigate whether the duration of uniocularity may play a more prominent role in determining the performance of uniocular individuals who are habitually involved in occupations that require fine depth discrimination (e.g. tailors, watchmakers, and goldsmiths).^56^

### Larger Head Movements but Limited Utility to Dynamic Visuomotor Task Performance

The uniocular individuals made sizeable head movements during the buzz-wire task that generated suprathreshold level of disparity signals from motion parallax (Table 5, Fig. 7). These head movements were also larger than those made by binocular controls (Table 5). Despite this, the results revealed no additional benefit of head movements in improving the buzz-wire task performance relative to the head-restricted condition (Table 4). This result is in alignment with those of Marotta et al.^21^ who found that larger head movements offered no additional benefit to hand reaching actions amongst uniocular individuals. Three reasons may be considered for this surprising result. First, the need to continuously move the hoop around the wire in the task requires a dynamic estimation of distance, depth, and curvature information, and modifying the visuomotor actions accordingly to avoid errors in the task. Perhaps the monocular cues to depth are not employed for such complex dynamic computations and may function better for static depth estimates.^13^ Adding motion parallax in the mix may only complicate the viewing scenario, for this cue derived from one form of motion action (head movements) should be updated dynamically and temporally synchronized to drive another form of motor action (hand movements). Second, extraretinal cues from head position^28^ and eye movements^57^^–^^59^ are critical for disambiguating the sign of depth derived from motion parallax. Stabilizing the head position during a depth from motion task or placing it in conflict with head velocity results in a significant weakening of the depth information derived from the motion parallax cue.^59^^,^^60^ Given that participants had to pay keen attention to the location of the hoop with respect of the wire, they may not have made many eye or head movements during the buzz-wire task, resulting in an ambiguous depth information from motion parallax. Third, unlike a typical motion parallax task where the object is stationary in space, both the object of regard (the hoop) and the head are in constant motion in the buzz-wire task. The visual system therefore has to disambiguate retinal image motion arising from head velocity from that arising from the velocity of object motion. This ambiguity, while possible to resolve,^61^^,^^62^ may be challenging enough to impair the depth calculations in a dynamic visuomotor task like the buzz-wire used here.

Taken together, although the perception of depth may be benefitted by the motion parallax cue derived from head movements, it may not benefit complex and dynamic visuomotor tasks engaged by humans as a part of their daily living activities. As an alternate possibility, the head movements made by participants in such visuomotor tasks may very well be a strategy to maximize the field of view of objects in the absence of binocularity (Appendix 2 Videos A2 and A3). All these complications exist even under binocular viewing conditions, but the visual system may effectively veto the information provided by the monocular depth cues in favor of the binocular disparity cue to determine task performance.

### The Role of Proprioceptive Feedback in the Buzz-Wire Task Performance

In addition to the visual cues, the proprioceptive pull/pressure of the hoop's contact with the wire may provide useful feedback to the brain about whether an error is made or not. In fact, several participants in the monocular/uniocular viewing conditions of present study described this sensation as a “magnetic force” preventing a break in contact between the hoop and the wire, regardless of their effort to disengage this contact. The complexity of the motor navigational operation and the associated proprioceptive feedback perhaps also explains why the proportion of contact between the hoop and the wire were significantly higher in the curved regions of the buzz-wire track, relative to the straight regions (Fig. 6). Although proprioceptive information is inherent to the buzz-wire task, this cue is unlikely to have dominated performance in this study. Should this have happened, the binocular and monocular task performance of controls would have been identical, and the task would have been deemed unfit for investigating the questions raised in the present study.

## Supplementary Material

Supplement 1

Supplement 2

Supplement 3

## Appendix I. Appendix I. Repeatability of Error Rate and Speed Across the Three Buzz-Wire Trials

Every study participant performed three variants of the buzz-wire task. This allowed a determination of the test-retest variability of the task performance. Figure A1 shows Bland-Altman plots for the error rate (panel A) and speed (panel B) for the binocular and monocular viewing of controls and for the uniocular participants. These data showed no bias across the three repetitions of the task (mean unsigned difference for error rate: <0.05 errors/sec; mean unsigned difference for speed: <0.15 cm/sec). The difference in error rate and speed across any two repetitions showed no systematic trend with the mean value across these repetitions (Figs. A1A, A1B). The 95% limits of agreement of the error rates and speed were in the same range among the three viewing conditions tested (Figs. A1A, A1B). Finally, the intraclass correlation coefficients revealed a good reliability of error rate (binocular = 77.7%; monocular = 79.5%, uniocular = 77.8%) and speed (binocular = 82.7%; monocular = 81.8%; uniocular = 79.1%) across the three repetitions. These results indicate no systematic difference in task performance across the three repetitions. Therefore the outcome measures obtained from these three repetitions were averaged for further analyses.

## Appendix II. Appendix II. Representative Face Tracking Videos

The head movements of the study participants were analyzed using a face tracking software (OpenFace, CMU School of Computer Science, Tech). This appendix shows representative videos of head movements made by a control participant under binocular (Video A1) and monocular (Video A2) viewing conditions and by a uniocular participant (Video A3). These videos were taken while the participants performed the bent buzz-wire task without any head restraint. The high-pitched audio buzz arising from the contact between the hoop and the wire, was analyzed with the Audacity software to generate the primary outcome variables of this study (Fig. 1D). The associated graphs show changes in the translational (horizontal, vertical and anteroposterior) and the rotational (pitch, yaw, and roll) head movements for each frame of the video, normalized to the head position during the first 5 seconds where the subjects looked directly at the camera.  Table A1 describes the sign convention for the head movements depicted in the videos. Both the controls under binocular and monocular viewing condition and the uniocular participant was noted to adopt a preferred head position as soon as the task started. Also, the head movements made during the task were not correlated to the locations where the hoop came in contact with the wire. Maximum errors under monocular and uniocular viewing occurred at the transition regions of the buzz-wire, wherein participants tried to turn the hoop, much ahead of the time and made a contact with the wire.

**Table A1.** Sign Conventions Indicating the Direction of Head Movements in the OpenFace Software Output

Head Movement	Positive	Negative
Translational		
Horizontal	Left side	Right side
Vertical	Downward	Upward
Anteroposterior	Farther	Closer
Rotational		
Pitch	Down head movement	Up head movement
Yaw	Right head turn	Left head turn
Roll	Right head tilt	Left head tilt
